# Research progress of gut microbiota and frailty syndrome

**DOI:** 10.1515/med-2021-0364

**Published:** 2021-10-12

**Authors:** Xiao Wang, Min Wu

**Affiliations:** Geriatrics Department, Zhejiang Hospital, Hangzhou 310013, China

**Keywords:** frailty, gut microbiota, chronic inflammation, neurodegenerative diseases, inflammatory factors

## Abstract

Frailty is a clinical syndrome caused by homeostasis imbalance. It is characterized by marked vulnerability to endogenous or exogenous stressors, reduced self-care ability, and increased mortality risk. This aging-related syndrome is common in individuals older than 65 years and carries an increased risk for poor health outcomes. These include falls, incident disability, incapacity, and mortality. In addition, it can result in a poor prognosis for other comorbidities. With the aging population, frailty increases the burden of adverse health outcomes. Studies on frailty are at their infancy. In addition, there is a lack of thorough understanding of its pathogenesis. Several studies have suggested that frailty is caused by chronic inflammation due to enhanced intestinal permeability following gut microbiota imbalance as well as pathogen-related antibodies entering the circulation system. These result in musculoskeletal system disorders and neurodegenerative diseases. However, this assumption has not been validated in large cohort-based studies. Several studies have suggested that inflammation is not the only cause of frailty. Hence, further studies are necessary to extend our understanding of its pathogenesis. This review summarizes the research findings in the field and expands on the possible role of the gut microbiota in frailty syndrome.

## 1 Frailty syndrome

Frailty syndrome is an aging-related syndrome and is characterized by morphological and physiological changes in multiple organs and systems. This leads to homeostasis imbalance and marked vulnerability to endogenous and/or exogenous stressors [1,2]. At present, frailty is clinically defined with frailty phenotype (FP) and frailty index (FI) (Table 1).

**Table 1:** Common frailty assessment models [4]

Frailty assessment models	Type	Contents	Author
Frailty phenotype (FP)	Criteria	Criteria: weight loss, weakness, slowness, low activity levels, poor endurance	Fried, 2001
		≥3 met: frail	
		1–2 met: pro-frail	
		0 met: non-frail	
Frailty index (FI)	Index	Calculating the proportion of abnormal items to the total items (FI value)	Mitnitski, 2001
		FI ≥ 25%: frail	
		8 < FI < 25%: pro-frail	
		FI ≤ 8%: non-frail	

Frailty syndrome has been highly associated with age. A meta-analysis [3] based on 21 studies demonstrated that the incidence rate of frailty syndrome ranges between 4 and 59.1% and is positively correlated with age. The incidence peak occurs in populations aged 85 years. Along with increased longevity and the higher proportion of the elderly in the population, the number of individuals with frailty syndrome keeps increasing [4]. Studies have demonstrated that frailty is closely related to chronic diseases, such as cardiovascular diseases, Alzheimer’s Disease (AD), and Parkinson’s Disease (PD) [4,5,6]. The prevention and treatment of frailty may reduce the risk of other chronic diseases and improve prognosis. Hence, understanding the pathogenesis and prevention of frailty may lower the burden of adverse health outcomes in the elderly. It is an important public health concern to understand the cause and pathogenesis of frailty.

## 2 Gut microbiota

Higher organisms contain diversified microflora, which includes, bacteria, archaebacteria, viruses, fungi, and protozoa [7]. The intestinal tract of mammals is rich in nutrition and has a constant temperature, making it ideal for the survival of microorganisms. Microorganisms reside in all mucosal surfaces of the host but are mainly distributed in the gastrointestinal tract. Most microorganisms are anaerobes outnumbering aerobe and facultative anaerobes by 100 to 1,000 times [8]. A sequencing analysis on the gut microbiota of a cohort of 386 Chinese individuals indicated that 95% of the gut microbiota were Firmicutes, Bacteroidetes, Proteobacteria, and Actinobacteria, of which, 90% fell into 15 genera, such as Bacteroidetes, Clostridia, *Clostridium leptum*, and Eubacteria [9].

There are approximately 10^14^ [10] bacterial cells in the intestinal tract of an adult. This is 10 times as much as the number of human cells [11]. Their combined genome (also known as the microbiome) tops over 5 million genes, two orders of magnitude higher than the genetic potentiality of the host [7,12]. The consequent huge gene product library (e.g., RNA and protein) exerts an influence on the physiological activities of the host [13]. Studies have shown that approximately 400,000 of the 4,026,600 messenger RNAs in the human transcriptome are from gut microbiota [14]. This lays a material basis for the gut microbiota to participate in regulating the physiological activities of the host: (1) Gut microbiota can stimulate the metabolism of polysaccharides [14], synthesize essential vitamins, and regulate fat absorption and distribution [10]. (2) The microbiota is essential for the development and differentiation of intestinal epithelial cells of the host. In addition, they can facilitate the maturation of gut-associated lymphoid tissues (GALTs), tissue regeneration (especially intestinal villi), and intestinal tract movement. (3) The microbiota plays a prominent role in shaping the immune microenvironment. This is done by promoting the development of lymphatics and the differentiation of immunocytes and regulating the generation of immune mediators [14]. In addition, they can stabilize the immune system of the host. (4) Gut microbiota has also been shown to regulate tissue homeostasis (e.g., induces cell proliferation and stem cell differentiation) and bone mineral density (BMD) of the host [14] and (5) the microbiota can affect the nervous system of the host through three pathways of the gut–brain axis, i.e., immune system, neuroendocrine system, and vagus nerve. This is important for modifying and controlling cognitive activities such as anxiety, pain, and depression [15].

The gut microbiota is influenced by the host and under dynamic fluctuations. Studies on the gut microbiota in children and adults suggest that the gut microbiota keeps changing from birth to old age [16,17]. After 65 years of age, the gut microbiota enters a degeneration period with decreased bacterial diversity and is usually dominated by Bacteroidetes [18]. Furthermore, changes in diet, lifestyle, sanitary conditions, or antibiotic use of the host could also affect the gut microbiome composition [14,19,20,21]. For instance, Firmicutes/Bacteroidetes ratio has been generally accepted as an index for obesity [22]. Unlike the genome of the host, the microbiome changes rapidly along with changes in gut microbiome composition or a single microbial gene. This leads to changes in the transcriptome, proteome, and metabolic profiling [14], and influences the host. Thus, alterations in the gut microbiota may exert a profound impact on the health of the elderly [15,23].

## 3 Direct evidence of correlation between gut microbiota and frailty

An imbalance in gut microbiota may trigger a chronic inflammatory status [22] and increase the risks of cardiovascular diseases, Type II diabetes, and cancer [24,25,26]. As studies on frailty syndrome increase, researchers have identified that gut microbiota imbalance in the elderly may be associated with frailty [27]. Nevertheless, there are only limited studies on the gut microbiota and frailty. A small number of exploratory studies have revealed a possible correlation [15,24].

### 3.1 Potential correlation between frailty and intestinal microorganism diversity

It is reasonable to believe that there is a correlation between frailty and intestinal microorganism diversity. The gut microbiota in the elderly is characterized by reduced bacterial community diversity and an increase in some microorganism species [5,28,29,30,31]. A small-scale exploratory study compared the gut microbiotas of individuals with and without frailty. The study discovered that Lactobacilli is decreased in frail people compared with non-frail ones, and the same thing happens in *Faecalibacterium prausnitzii* (with anti-inflammatory and immunoregulation effects); while Enterobacteriaceae, which could increase opportunistic infections [32], was found to be higher in frail people. Another study performed on 728 female twins suggested a negative correlation between frailty and intestinal microorganism diversity [29]. *Eubacterium dolichum* (related to a high-fat diet [33]) and *Eggerthella lenta* (a pathogen [34]) are the most abundant gut microbiota in individuals with frailty. Like that of the elderly, *F. prausnitzii* in the gut microbiota of individuals with frailty decreased [35].

Studies using larger cohorts are necessary to determine whether the gut microbiota is associated with frailty. Additionally, it is necessary to perform high-quality clinical trials on intervention treatment and assess the effects of probiotics supplements and/or prebiotics on frailty syndrome. A randomized double-blind placebo-controlled clinical trial investigated interventional treatments [36]: 60 volunteers received a 13-week treatment with probiotic preparations (inulin and fructo-oligosaccharide). Subsequent frailty assessments demonstrated that frailty was not improved. Nevertheless, compared to the placebo group, individuals receiving probiotic treatment had significant improvements in two indicators (i.e., fatigue and grip).

### 3.2 The gut microbiota and frailty may influence each other

In addition to the potential correlation, the gut microbiota and frailty may influence each other. The microbiota may be possibly affected by drugs taken by patients with frailty, especially proton pump inhibitors (PPIs), which are prescription drugs administered to older individuals with frailty [37,38,39]. A large population-based study on frailty demonstrated a decrease in symbiotic bacteria abundance and bacterial diversity and increased levels of pathogenic streptococci in the intestinal tract of patients taking PPIs. These results were validated in a study on fraternal twins taking PPIs [40]. These findings are of clinical significance because it proves that the use of PPIs and alterations in the gut microbiota were related to *Clostridium difficile* infection (CDI). CDI may give rise to a poor prognosis and frailty in elderly patients with multiple complications [41]. In addition, non-steroid anti-inflammatory drugs (NSAIDs) that have been extensively administered in the elderly may lead to alterations in the gut microbiota [42].

## 4 Role of gut microbiota in the pathogenesis of frailty

The above studies revealed a direct correlation between the gut microbiota and frailty. Adverse outcomes brought by aging may include alterations in the gut microbiota [43,44] and an inflammatory reaction (immunosenescence) [45], followed by frailty. Studies have asserted that the gut microbiota may play a part in the pathogenesis of frailty through chronic inflammation [5]. However, the relationship with regards to the alterations in the gut microbiota, chronic inflammation, and frailty is not unilateral but complicated and interrelated [46,47]. The mutual influences among alterations in the gut microbiota, chronic inflammation, and frailty will be discussed in the following sections.

### 4.1 Frailty syndrome and immune-related disease in the context of aging

As mentioned above, frailty syndrome leads to homeostasis imbalance and marked vulnerability to endogenous and/or exogenous stressors [1,2]. For example, it is well studied that frailty syndrome is always with a chronic low-grade inflammation, which is a contributing factor of immune related disease [48]. So, it is reasonable to believe that the elder population with frailty syndrome is highly like with immune-related diseases.

The clinical manifestations of frailty syndrome are usually loss of muscle and bone tissue, which may partially be explained by an impaired immune system as studies by Cornish et al. who revealed that there is substantial “cross-talk” between muscle and bone and the immune system [49].

Stavropoulou and Bezirtzoglou found that the elder population with frailty is always the one facing immune-related disease. Usually, fragile elder population is facing problems like changes in hormonal, increase in the pro-inflammatory cytokines release, abnormality of the telomere, and these problems can cause the dysfunction of the immune system [50].

### 4.2 Gut microbiota, chronic inflammation, musculoskeletal system disorders (MSDs), and frailty

A possible cause may be aging-triggered alterations in the gut microbiota which results in chronic inflammation. Increased inflammatory factors could directly or indirectly lead to typical symptoms of frailty like reduced grip and muscle and bone loss [51,52].

Studies have demonstrated that changes in gut microbiome composition and intestinal permeability due to aging may give rise to an inflammatory reaction. Studies have revealed that aging is associated with decreased probiotics (e.g., *Enterococcus faecalis*, *Bacillus faecalis*, and Lactobacilli) and a lower Firmicutes/Bacteroidetes ratio, despite the tremendous differences in the gut microbiota of populations of different races and in different regions and environments [53].

Some beneficial microorganisms are vital to human health because they can inhibit the expansion of pathogenic bacterial communities, and generate mucus and products of lipid metabolism, for example, short-chain fatty acid (SCFA), bacterial polysaccharide (PSA), and Serum Amyloid A (SAA), through fermenting starch and dietary fiber to maintain a complete intestinal tract barrier [54]. It has been reported that SCFA can act on internal regulatory T cells (cTreg) through the G protein-coupled receptor GPR43 to improve the number of cTregs which is reduced by vancomycin, and up-regulate the genes of Foxp3 and IL-10 in cTreg cells in sterile mouse, so as to alleviate colitis [55]. Another research shows that SCFA can increase the immunoglobulin A (IgA) level in rat saliva, thus revealing a possible mechanism of how cellulose enhances rats’ immunity [56].

Intestinal beneficial bacteria decrease with age, while the relative abundance of other bacteria increases, including symbiotic bacteria that are pathogenic and inflammatory [57]. Such microorganisms are mainly facultative anaerobes (e.g., Clostridia and Staphylococci). In addition, studies have demonstrated that an increase in pathogenic bacteria is associated with an increase in inflammatory cytokines [58]. Enhanced intestinal permeability enables bacteria and their products (including pathogen-associated molecular pattern (PAMP), damage-associated molecular pattern (DAMP), and microorganism-associated molecular pattern (MAMP)) to enter the circulatory system. This consequently results in a chronic pro-inflammatory status [59]. This hypothesis is supported in animal models, however, no explicit evidence has been identified in the elderly who have no obvious inflammatory disease [60].

An inflammatory reaction manifests with an increase in inflammatory factors (e.g., IL-6, C-reactive protein, tumor necrosis factor-α (TNF-α), and neopterin) [61]. Several studies have demonstrated that high levels of inflammatory molecules in the blood have been correlated with frailty [62]. Hence, chronic inflammation attributable to alterations in the gut microbiota may be a key cause of frailty.

Studies have demonstrated the direct correlation between increased inflammatory factor levels and frailty [45,46,47,51,52,53,54,57,58]. The direct correlation between frailty and elevated IL-6 levels (a pro-inflammatory cytokine) was demonstrated in a study that involved community-dwelling elderly individuals [63]. The IL-6 level in the serum of individuals with frailty was higher compared to individuals without frailty. This exploratory study enrolled 11 senior citizens with frailty and 19 without frailty. This finding was validated subsequently in a large-scale study in elderly individuals under different nursing conditions, as well as in cell culture and mouse model-based studies. This suggested a direct correlation between chronic inflammation and immune activation, characterized by elevated IL-6 levels and frailty syndrome [64]. Additional inflammatory molecules (e.g., C-reactive protein and TNF-α) were also demonstrated to be related to frailty syndrome [65]. In addition, increased neopterin level (a marker of immune activation), independent of IL-6 levels, were confirmed to be associated with frailty in a cohort of community-dwelling elderly individuals [66]. These indicated that immune activation may induce frailty relative to chronic inflammation [67]. An increase in total white blood cell counts (TWCC) (higher than typical ranges) is often regarded as part of the complete blood counts for routine measurement in clinical practice. This is a laboratory index secondary to systemic inflammation induced by acute bacterial infections. Several studies have demonstrated a direct correlation between increased TWCC (even within the normal range) and frailty. Specific subsets, including neutrophils and monocytes, have been confirmed to be associated with frailty [68]. In addition, an increase in other cell subsets, such as differentiated CD8+/CD28− T cells and CCR5+ T cells, has also been associated with frailty [69,70,71].

As previously mentioned, the direct correlation between frailty and inflammatory molecules has been demonstrated. Higher expression levels of inflammatory molecules activate the inflammatory pathway, and it is a molecular mechanism of chronic inflammation in individuals with frailty [72]. How does chronic inflammation play a role in the pathogenesis of frailty? It was discovered that several inflammatory molecules (e.g. IL-6) may directly induce frailty or reduce key assessment indicators (e.g., muscle mass, strength, and exercise performance) [73,74]. A study on 3,075 elderly individuals demonstrated that high levels of IL-6 were linked to reduced muscle mass and strength. The relationship between the IL-6 level and grip has been the most consistent. The increase in IL-6 level by each standard deviation (SD) results in a decrease in grip by 1.1–2.4 kg [61]. Studies have demonstrated that higher levels of inflammatory molecules were negatively related to hemoglobin levels and levels of insulin-like growth factor-1 (IGF-1), albumin, micronutrients, and vitamins [63,75,76]. IGF1, in particular, is an indispensable growth factor for muscle regeneration and muscle structural integrity and protects the body from unstable carotid atheromatous plaques [77,78]. *In vitro* studies showed that IL-1, IL-6, and TNF-α could inhibit IGF1-mediated anabolism. In addition, IL-6 reduces the generation of IGF1 and IGF-binding protein 3 (IGFBP-3) [79]. An observational study suggested that high levels of IL-6 and low levels of IGF1 could exert a synergistic effect to decrease muscle strength and effectively predict progressive disabilities and death [80,81]. Furthermore, inflammation interferes with long-chain peptide synthesis which is indispensable for muscle energy and protein anabolism [82].

**Figure 1:**
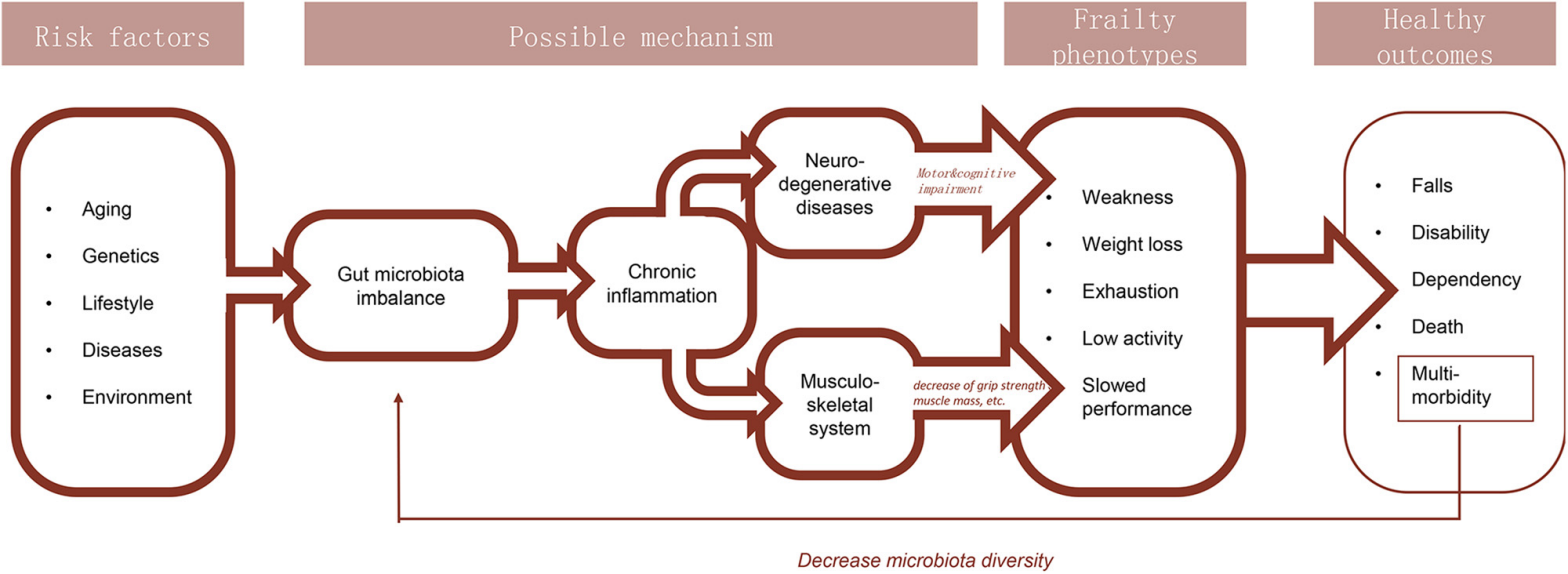
Current understanding of the pathogenesis of frailty syndrome.

Chronic inflammation directly or indirectly plays a key role in the pathogenesis of frailty (as shown in Figure 1). Chronic inflammation causes typical symptoms of frailty, such as reduced grip and muscle mass by damaging the musculoskeletal system. In addition, other factors besides chronic inflammation may play a vital role in the pathogenesis of frailty. Some studies found no correlation between elevated IL-6 levels and frailty [83,84], while other studies found that administration of statins that had an anti-inflammatory effect did not alleviate frailty [85]. Given this, the pathogenesis of frailty is extremely complicated. Chronic inflammation is probably one of the causes. Furthermore, the gut microbiota may influence the host through anabolic resistance [86] and reduced bioavailability of nutrients [87]. Additional studies are necessary to identify causal factors for frailty.

## 5 Gut microbiota, neuroinflammation, neurodegenerative diseases, and frailty

Another possible cause of frailty may be related to gut microbiota. The mechanism may be through the gut–brain axis, which affects the nervous system to induce neurodegenerative diseases. This affects cognitive function to trigger or deteriorate frailty-related symptoms, including difficulty in moving and incapacity [37,88,89]. Such cognitive impairment-related frailty is known as “cognitive frailty” [90,91].

The concept of the gut–brain axis has been fully demonstrated and widely accepted. A study by Muller et al. [92] further verified this concept by discovering the direct modulation of gut-extrinsic sympathetic neurons by the gut microbiota. It is reported that the depletion of rat microbiota can induce an increase in the expression level of cFos, a neuronal transcription factor. The implantation of SCFA-producing bacteria rescues the increase of cFos expression, which further verifies that gut microbiota can directly regulate the development of these sympathetic neurons.

A large cohort study suggested a significant correlation between bacterial diversity and cognitive function [93]. This study sequenced fecal samples from 1,551 subjects and analyzed diversity to correlate with multiple cognitive functions, which included verbal fluency and response time. The authors observed a correlation between higher language diversity and shorter response time and higher bacterial diversity. There are only a limited number of studies on how gut microbiota imbalance impairs cognitive function and ultimately lead to frailty. However, several animal models and some studies on humans suggested a correlation between the gut microbiota and cognition [94,95,96,97].

Patients with Parkinson’s Disease (PD) suffer from shaking, stiffness, and difficulty in walking, balance, and coordination [98]. The primary pathogenesis lies in the unusual folding and aggregation of the protein α-synuclein, which forms protein clumps (i.e., Lewy bodies and Lewy neuritis) and affects the nervous system. Neuroinflammation induces gut–brain axis damage and is considered as a possible cause of abnormal folding of α-synuclein [99]. A study performed on 19 PD patients demonstrated that neuroinflammation may be triggered by chronic inflammation in the colonic mucosa [100]. Compared to healthy individuals, PD patients had higher mRNA expression levels of pro-inflammatory cytokines (e.g., TNF-α, IFN-γ, IL-6, and IL-1β) and neurogliocyte activation markers in colonic biopsies. It was observed that the levels of pro-inflammatory cytokines and the duration of the disease were negatively correlated. Another retrospective study indicated that vagotomy reduced the risk of PD. This suggested an interaction between the intestinal tract and the central nervous system [101]. Furthermore, it was observed that abnormally folded α-synuclein appears in the nerve plexuses of intestinal submucosa and myenteron before it aggregates in the brain. Hence, abnormal proteins may migrate to the brain from the intestinal tract in a “prion-like” form [102]. This assumption is supported by the cephalo-caudal gradient of the distribution of α-synuclein in the enteric nervous system (ENS) of PD patients during the early stages [103]. Hence, this could be the reason why PD patients suffer from gastrointestinal symptoms like constipation and dysporia several years before dyskinesia [104].

A PD mouse model (overexpressing α-synuclein) further revealed an important correlation between neuroinflammation and the gut microbiota. The comparison between germ-free and normal mice in this model indicated that the gut microbiota was indispensable to trigger dyskinesia, neurogliocyte activation (neuroinflammation), and aggregation of α-synuclein [105]. Additionally, the transfer of gut microbiota from PD mice (overexpressing α-synuclein) into germ-free mice resulted in sports injury, while transferring gut microbiota from healthy mice did not.

Human studies have demonstrated the correlation between the gut microbiota and PD. A study assessed the gut microbiome composition of 72 PD patients: [106] Compared to healthy subjects, Prevotellaceae in the feces of PD patients were significantly lower, while the relative abundance of Enterobacteriaceae increased. This was positively correlated with the severity of postural instability and gait disturbance (PIGD) of patients. The authors speculated that Prevotellaceae could significantly increase the synthesis of thiamine and folic acid to generate mucoprotein. Hence, a decrease in Prevotellaceae may contribute to reduced vitamin content and enhanced intestinal permeability observed in PD patients.

Alzheimer’s Disease (AD) is the most common type of dementia. The disease process is associated with amyloid plaques and neurofibrillary tangles in the brain [107]. The correlation between the intestinal tract and AD was first demonstrated in a mouse model. Compared to conventionally fed mice with a complete gut microbiota, germ-free mice had memory dysfunction (a typical symptom of AD) [108]. Moreover, the administration of endotoxin could increase β-amyloid protein levels in the hippocampus of mice and induce cognitive defects [109]. An interventional study demonstrated that higher numbers of Actinomycetes and Bacteroidetes [110] decreased neurogliocyte activation markers and enhanced brain-derived neurotrophic factor (BDNF) in the gut microbiota of older rats treated with VSL#3 (a probiotic mixture of eight Gram-positive bacterial strains).

Animal models have suggested the potential relationship between AD and gut microbiota [102,103,104], however, this relationship has only been supported by a limited number of research studies on humans with AD. Microbiota in the feces of AD patients was first analyzed in a study conducted in 2017 [111]. In this study, compared to the control group and patients negative for amyloid protein, patients who were positive for amyloid proteins had more pro-inflammatory cytokines (e.g., IL-6, CXCL2, NLRP3, and IL-1α) and lower levels of IL-10, an anti-inflammatory cytokine. In terms of the gut microbiota, the level of *Eubacterium rectale* in patients who were positive for amyloid protein was lower, while the levels of *Escherichia* and *Shigella* that cause infections were increased. In addition, it is found that pro-inflammatory cytokines (e.g., IL-1α, NLRP3, and CXCL2) were positively correlated with the abundance of *Escherichia* and *Shigella*, and negatively correlated with *Bacillus stearothermophilus* level in the rectum.

**Figure 2:**
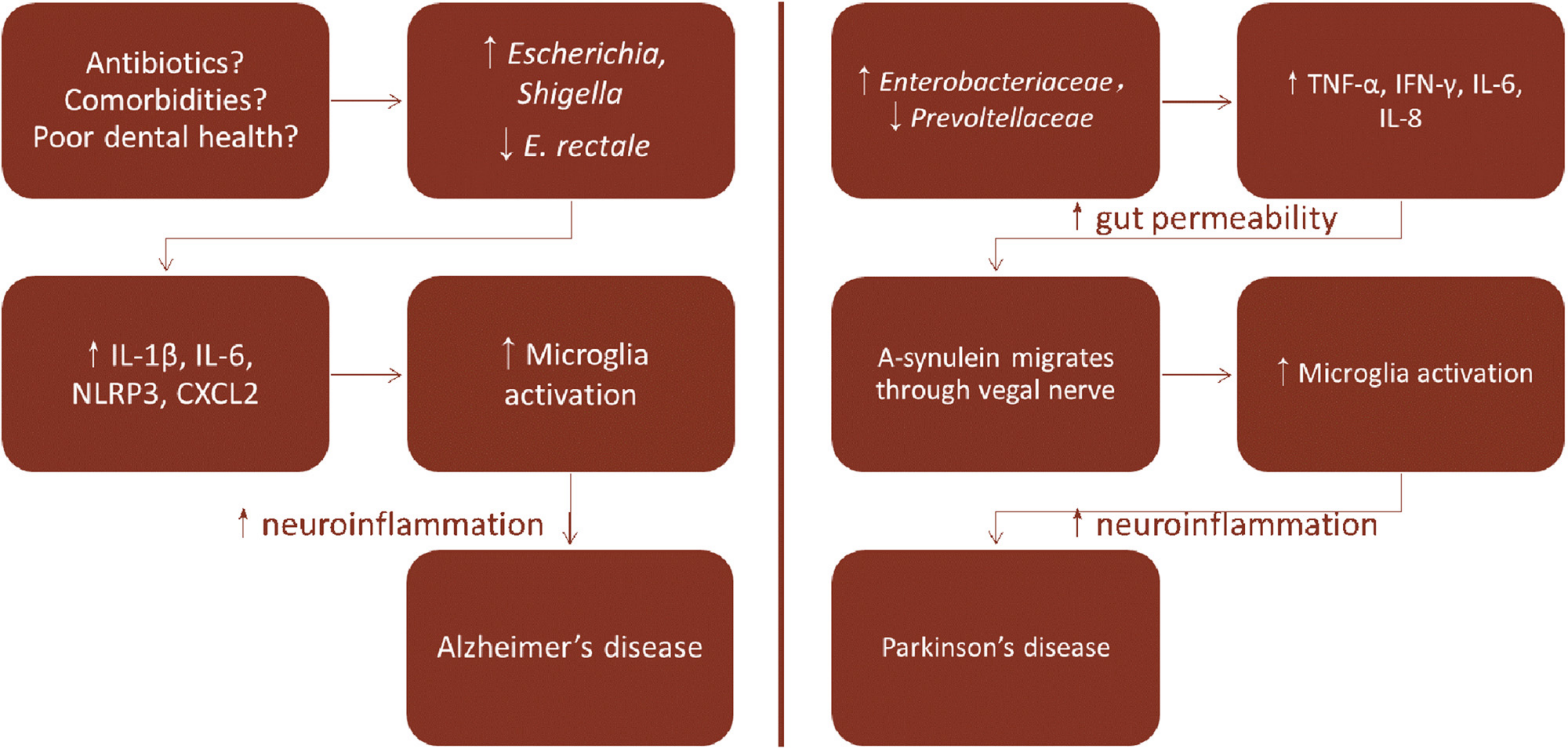
Possible pathogenic mechanism of PD and AD due to gut microbiota imbalance [5].

Animal models and some human studies have demonstrated the relationship between chronic inflammatory status triggered by gut microbiota and the marked upregulation of immune factors and neuroinflammation characterized by neurogliocyte activation. Hence, some investigators speculated that the upregulation of inflammatory factors, due to gut microbiota imbalance, leads to neuroinflammation through the gut–brain axis. Subsequently, neuroinflammation takes part in the onset/progression of PD and AD [5] (as shown in Figure 2). Dyskinesia and cognitive disorders brought about by PD and AD will cause/deteriorate symptoms of frailty, which include slower movement and reduced physical activity, lower the self-care ability and increase the risk of death [37]. Nevertheless, the assumption has not been fully demonstrated in human studies. How neuroinflammation participates in the pathogenesis of PD and AD remains to be deciphered.

Apart from the impact on central nervous system, the enteric neurons are under the regulation of the gut flora as well. Take the association between IL-33 and enteric neurons for an example. Malik et al. [112] observed a higher level of pro-inflammatory microbiota in mice lacking IL-33, which indicated a direct association between IL-33 level and gut microbiome. On the other hand, the IL-33 level is associated with the release of 5-HT. IL-33, as an alarmin cytokine, could be sensed by Enterochromaffin (EC) cells [113], resulting in the release of serotonin (5-HT) [114], a neurotransmitter that activates enteric neurons and promotes gut motility, which is essentially compromised in frail people [115]. Therefore, it is reasonable to relate gut microbiota-related IL-33 increase with 5-HT decrease-related frailty symptoms.

## 6 Discussion and conclusion

As the population ages, frailty syndrome will bring a huge medical burden to society. Previous studies have suggested that gut microbiota imbalance may be a cause of frailty. Animal models and a few human studies have demonstrated that individuals with frailty tend to have increased levels of inflammatory factors (e.g. IL-6, C-reactive protein, and TNF-α) and a chronic inflammatory status. Inflammatory factors have been demonstrated to directly or indirectly reduce key indicators of frailty, such as muscle mass and grip. In addition, gut microbiota imbalance has been demonstrated to be associated with the higher expression of inflammatory factors. Studies have suggested that gut microbiota imbalance leads to enhanced intestinal permeability. This in turn triggers the entry of pathogen-related antibodies like PAMP and DAMP to the circulatory system to subsequently trigger an inflammatory reaction. As a result, investigators believe that the chronic inflammatory status due to gut microbiota imbalance could directly or indirectly give rise to the typical symptoms of frailty (by causing cardiovascular diseases or damaging the musculoskeletal system). In addition, higher levels of inflammatory factors due to gut microbiota may further influence the nervous system of the host via the gut–brain axis to induce neuroinflammation (neurogliocyte activation) leading to neurodegenerative diseases, i.e., dyskinesia and/or cognitive disorders in patients with frailty (as shown in Figure 1).

However, the above assumptions have not been validated in large cohort-based studies. The relationship of gut microbiota imbalance, chronic inflammation, and frailty is not unilateral but complicated and interrelated. Several studies have suggested that chronic inflammation due to gut microbiota imbalance may not be the only cause of frailty. It is worth noting that individuals with frailty are on long-term medication due to preexisting chronic diseases (complications). It has been demonstrated that medications could alter gut microbiome composition. Hence, future studies are necessary to determine whether gut microbiota is a cause of frailty or a result of long-term medication in people with frailty. In addition, factors that may affect the gut microbiota, such as lifestyle, diet, and other health complications, need to be considered comprehensively. Lastly, studies on the pathogenesis of frailty should emphasize the prevention and treatment of frailty to improve the health and ease the medical burden of the elderly.

## Abbreviations

MSDs: musculoskeletal system disorders
FP: frailty phenotype
FI: frailty index
AD: Alzheimer’s disease
PD: Parkinson’s disease
GALTs: gut-associated lymphoid tissues
BMD: bone mineral density
PPIs: proton pump inhibitors
CDI: *Clostridium difficile* infection
NSAIDs: non-steroid anti-inflammatory drugs
PAMP: pathogen-associated molecular pattern
DAMP: damage-associated molecular pattern
MAMP: microorganism-associated molecular pattern
TNF-α: tumor necrosis factor-α
TWCC: total white blood cell counts
SD: standard deviation
IGF-1: insulin-like growth factor-1
ENS: enteric nervous system
PIGD: postural instability and gait disturbance
BDNF: brain-derived neurotrophic factor
